# Musculoskeletal Adverse Events Associated with PCSK9 Inhibitors: Disproportionality Analysis of the FDA Adverse Event Reporting System

**DOI:** 10.1155/2022/9866486

**Published:** 2022-01-25

**Authors:** Lingqing Ding, Congqin Chen, Yongkuan Yang, Jie Fang, Longxing Cao, Yige Liu

**Affiliations:** ^1^Department of Pharmacy, Xiamen Cardiovascular Hospital, Xiamen University, Xiamen 361024, China; ^2^School of Electrical Engineering and Automation, Xiamen University of Technology, Xiamen 361024, China; ^3^Department of Pharmacy, Ruijin Hospital, School of Medicine, Shanghai Jiao Tong University, Shanghai 200003, China; ^4^Department of Cardiovascular Medicine, Xiamen Cardiovascular Hospital, Xiamen University, Xiamen 361024, China

## Abstract

**Background:**

Some studies suggest that potential safety issues about PCSK9 inhibitors have not been sufficiently explored in clinical trials, including musculoskeletal adverse events (MAEs).

**Objective:**

To examine the association between use of PCSK9 inhibitors with and without concurrent statins and risk of MAEs. *Patients and Methods*. FDA Adverse Event Reporting System (FAERS) dataset of PCSK9 inhibitors and statins from October 2015 to June 2021 was queried. The reporting odds ratio (ROR) with relevant 95% confidence interval (95% CI) was calculated as the index of disproportionality. Outcome of MAEs of different PCSK9 inhibitors regimens was also investigated.

**Results:**

3,185 cases of PCSK9 inhibitor-associated MAEs were recorded. PCSK9 inhibitor class alone demonstrated a strong link to MAEs (ROR 5.92; 95% CI 5.70-6.15), and evolocumab was associated with more reports of MAEs than alirocumab. Concomitant use with statins leaded to an increased occurrence of MAEs (ROR 32.15 (25.55-40.46)), and the risk differed among different statins. The PCSK9 inhibitors were safer than statins in terms of hospitalization rate and death rate (15.64% vs. 36.83%; 0.72% vs. 3.53%).

**Conclusions:**

This pharmacovigilance investigation suggests that PCSK9 inhibitors are associated with MAEs. The risk significantly increases when combined with statins. Increased laboratory and clinical monitoring are required to timely diagnose and manage MAEs.

## 1. Introduction

For statin-intolerant patients, or those who have been prescribed with high-intensity statins but failed to reach their low-density lipoprotein cholesterol (LDL-C) target levels, it is especially difficult for them to gain therapeutic efficacy. One of the potential ways is the use of the recently approved proprotein convertase subtilisin/kexin type 9 (PCSK9) inhibitors, alirocumab and evolocumab. They have been proven to notably reduce LDL-C levels alone or on a background of statin therapy and are changing the therapeutic landscape of dyslipidemia [1, 2]. It has been a consensus that musculoskeletal adverse events (MAEs) were the well-described side effects of statin therapy which were dominated by myalgia, myopathy, and rhabdomyolysis, etc., although the underlying mechanism remains elusive that may be attributed to the novel immunogenetic factors, gender, etc. [3]. Extending the fear of MAE concept to PCSK9 inhibitors, most clinical trials evaluated MAEs as a safety end-point. Preliminary safety analyses suggested alirocumab and evolocumab had an acceptable safety profile and tolerability [4, 5]. The most common adverse events (AEs) were nasopharyngitis, upper respiratory tract infection, while MAEs were reported less frequently [6–8].

Despite low evidence, many researchers are still worried that MAEs have not been sufficiently explored in the clinical trials of PCSK9 inhibitors. In a phase 3 trial for alirocumab, ODYSSEY LONG TERM [9], the alirocumab group was seen with more cases of myalgia than the placebo group (5.4% vs. 2.9%). 281 subjects from the ODYSSEY ALTERNATIVE trial [10] entered an open-label treatment period (OLTP) [11], and received alirocumab for ∼3 years. The results indicated that the rate of MAEs in the OLTP (38.4%) was still high as that in parent trial (32.5%). From clinical trials to clinical practice, the published postmarket pharmacovigilance studies [12–14] have pointed out an association between PCSK9 inhibitors and muscle symptoms in clinical practice, either myalgia or joint-related-signs/symptoms. In addition, myalgia was a major reason for drug discontinuation in [12].

Besides, in terms of pharmacokinetics/pharmacodynamics (PK/PD), statins can induce the expression of the PCSK9 and thus increases the target-mediated clearance of alirocumab and evolocumab [15–18]. In an open-label extension trials [19], MAEs were more likely to affect patients in the evolocumab plus standard of care (SOC, statin, or ezetimibe) group than in the group of SOC alone (10.0% vs. 4.6%). So, the question arises whether in clinical settings, drug-drug interactions (DDIs) between PCSK9 inhibitors and statins have an effect on the risk of PCSK9 inhibitor-induced MAEs.

Unfortunately, real-world data on MAEs of PCSK9 inhibitors is limited. From a safety point, the rapid rise in the use of PCSK9 inhibitors coincides with the rise of the number of AEs; thus, it is worthy and urgent to address this controversial issue. In light of this, we utilize FDA Adverse Event Reporting System (FAERS) to update the safety data from clinical trials to real-world experience. Meantime, postmarketing surveillance can provide a crucial complement by presenting a real scenario where more than one drugs are prescribed and previously unanticipated DDIs of interest are signaled.

Based on the FAERS, this study is aimed at examining the association between use of PCSK9 inhibitors with and without concurrent statins and risk of MAEs. We further investigate the serious outcome rates for MAEs of different PCSK9 inhibitor regimens.

## 2. Methods

### 2.1. Data Source

Alirocumab was approved by FDA on 24 July 2015, and evolocumab was on 27 August 2015. So, we downloaded the data from the FAERS website from October 2015 to June 2021. The following reasons explain why the FAERS attracts our attention: collection of millions of spontaneous adverse events reports; public and easy access to raw data obtained in a format suitable for external researchers or consumers to look to signal detection of adverse drug events; published previous studies demonstrating great accuracy in detecting safety signals, especially for DDIs, and monitoring uncommon adverse events [20–22].

We managed the raw FAERS data in local by SQL server 2008 software (Microsoft SQL Server 2008 R2, Microsoft Corporation, Redmond, WA, USA). Prior to conducting any analysis, deduplication technique was applied to increase data quality. To increase reliability of results, we only gathered cases submitted by health professionals.

Given all the included reports in our study were presented by a unique primary ID, and all the data were gained from a public database, the study did not require informed consent and ethics.

### 2.2. Target Drug Identification

A list of drugs of interest was identified through exploration of MICROMEDX, FDA.gov, and Drugs.com. The drug names of the two approved PCSK9 inhibitors and seven approved statins are detailed in Supplementary Table 1. To reduce the risk of false-positive results, only drugs classified as “primary suspected” and “secondary suspected” were included in the analysis.

### 2.3. Target Musculoskeletal Adverse Event Identification

Referring to the Medical Dictionary for Regulatory Activities (MedDRA, version 24.1) and Standardized MedDRA Queries (SMQs), 65 MAE-related query preferred terms (PTs) are considered. The detailed adverse events are shown in Supplementary Table 2.

### 2.4. Serious Outcomes

Outcome is coded in the FAERS system. A report is designated as serious if an AE results in death (DE), life-threatening (LT), required hospitalization or prolongation of existing hospitalization (HO), disability (DS), congenital anomaly or birth defect (CA), required intervention to prevent permanent impairment/damage (RI), and other serious medical events (OT) [23]. The number and percentage of the reports with serious outcome in group of the signaled cases were calculated.

### 2.5. Data Mining

Since the aim of our study is to detect the possible occurrence of MAEs in patients exposed to PCSK9 inhibitors alone and further, compare the reports of PCSK9 inhibitors together with statins with the condition where they are prescribed alone; the target population is categorized into three groups [20]: reports of patients prescribed PCSK9 inhibitors and without statins; reports of patients prescribed statins and without PCSK9 inhibitors; reports of patients simultaneously prescribed PCSK9 inhibitors and statins. The reference group is composed of reports exposed to neither PCSK9 inhibitors nor statins.

From the mathematical point of view, the idea of case-noncase approach is to compare the proportion of an AE of interest exposed to a specific drug (cases) with the reports of the same reaction not exposed to this drug (noncases) [24, 25]. This so-called case/noncase approach can be considered as a case-control analysis, and their results can be measured using the reporting odds ratio (ROR) with their 95% confidence interval (95% CI). In our study, disproportionality was determined by ROR. ROR is calculated according to the formula: ROR = (Na∗Nd)/(Nb∗Nc), where Na is the number of the reports of a specific ADR for selected drugs; Nb is the reports for selected drugs without reporting this ADR; Nc is the number of the reports of a specific ADR for all other drugs; Nd is the number of the reports for all other drugs of interest without reporting this ADR. The signal was defined by the criterion of the lower limit of the 95% CI of ROR > 1 and the number of reports > 3.

## 3. Results

### 3.1. Demographics

During the about 6-year period, a total of 50,213 cases and 3,867,797 noncases were identified. Within these reports, 3,088 cases were associated with PCSK9 inhibitors alone, 7,817 cases with exposure to statins alone, and 97 cases with exposure to the two combined drugs. With regard to the PCSK9 inhibitor regimen groups, median age was 68 years (interquartile range 60-75) and the majority of reports involved patients aged >45years, with slight female preponderance (54.44% vs. 40.09%). Physicians were the predominant source of reports (72.65%). The USA ranked first among the reported countries (95.65%), followed by Germany (0.73%). Notably, the number of reported cases of PCSK9 inhibitor regimens fluctuated without an apparent trend, especially a peak in 2018, while reports of statins appeared an increasing trend over years. Additional demographic information can be found in Table 1.

### 3.2. Adverse Event Signals

Table 2 provides detailed RORs and their 95% CI for two drugs separately and together used. To control the effect of a reported concomitant stains on the occurrence of MAEs and to better display true disproportionality, cases were further filtered. Cases exposed to statins which was coded only as “concomitant” role instead of “primary suspected” and “secondary suspected” role were removed from the analysis to prevent inadvertent overestimation of ROR. We identified a statistically significant disproportionality signal of muscle symptoms for PCSK9 inhibitors without statins (ROR 5.92(5.70-6.15)). Evolocumab was the most frequently reported PCSK9 inhibitor, but its MAE risk was lower than alirocumab (ROR 5.59 (5.37-5.81) vs. ROR 9.79 (8.83-10.85)). It is noted that statin monotherapy showed a higher risk of MAEs (ROR 20.17 (19.64-20.70)) as compared to PCSK9 inhibitor monotherapy. An apparently increased risk was found for PCSK9 inhibitors combined with statins (ROR 32.15 (25.55-40.46)), higher than that found for the two drugs used alone. The risk differed when used with different statins, and the statins were as follows in ascending order of risk: atorvastatin < rosuvastatin < simvastatin < pravastatin < pitavastatin.

### 3.3. Outcomes

For PCSK9 inhibitor regimens, only 17.60% records were reported with outcomes, among which the hospitalization rate was 31.54%, and death rate was 7.36% as Table 1 shows. For cases of MAEs, the outcome distribution in terms of each PT is shown in Table 3. The hospitalization rates of cases with MAEs were similar across different PCSK9 inhibitors regimens (15.64% for PCSK9 inhibitors alone and 17.58% for concomitant with statins). To note, the outcomes for statins associated with MAEs was not good, with a 36.80% hospitalization rate and a 3.53% death rate.

## 4. Discussion

To our knowledge, our study is the first and the most comprehensive attempt so far to evaluate the risk of PCSK9 inhibitor-associated MAEs in the real-world practice based on the FAERS. This study extends the previously published pharmacovigilance information in more accurate ways: limiting the selected reports submitted by physician report source; analyzing reports in which the drugs were coded as “primary suspected” and “secondary suspected.”

### 4.1. Demographic Information

In our study, PCSK9 inhibitor-associated MAEs appeared to influence more females than males (54.44% vs. 40.09%). This finding is consistent with another pharmacovigilance investigation of Lareb and vigilyze [12]. Subjects aged 45-64 years and 65-74 years were similarly affected. Besides, it needs our attention; there were neonates (e.g., 62 days and 85 days after birth) reported with PCSK9 inhibitor-associated MAEs in our study, although HAUSER-RCT trial [26] did not detect the muscle symptom adverse effects among pediatric patients. Thus, the long-term safety and efficacy of PCSK9 inhibitors in the pediatric population will require continued study.

**Figure 1:**
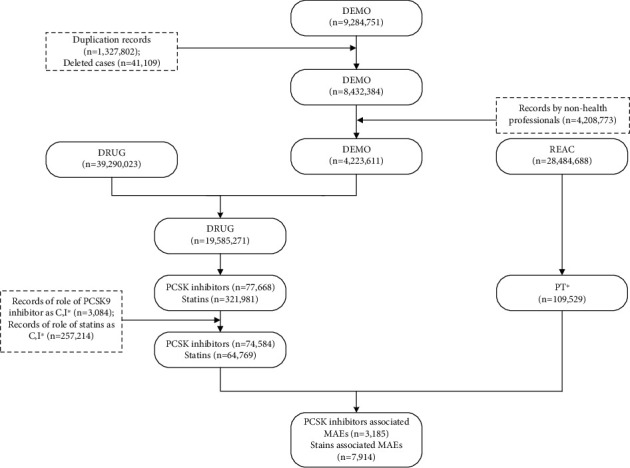
Flowchart of identifying cases with musculoskeletal adverse events reported by health professionals from FAERS database.

A unique finding was that the number of noncase reports of statin monotherapy was less than that of PCSK9 inhibitor monotherapy. It is out of our expectation, since the first-in-class statin was approved by FDA at 1987 while the two PCSK9 inhibitors were introduced into market from 2015. The resultant statins account for only 20.12% in the initial reports as Figure 1 shows, yet PCSK9 inhibitors up to 96.03%. The possible reason behind the large gap mainly lies in that we only focus on reports submitted by health-care professionals and reports coded with suspected drugs. This phenomenon highlights the existence of nocebo effects of statins among consumers, which is described in detail later.

### 4.2. PCSK9 Inhibitor-Associated MAEs

Some studies [27–29] have pointed out that the pharmacological effect of statins is not necessarily involved with the development of statin-associated MAEs since the number of reports of statin-associated MAEs in observational studies was higher than that in RCTs. In this context, the term nocebo effect, which is regarded as negative expectations that contribute to negative consequences, needs to be considered [30]. Increased publicity, patients' knowledge of the potential for adverse events of statins therapy, and verbal suggestions of symptoms may be the factors contributing to the experience of nocebo effects of statins. Subsequently, nocebo effects of statins may potentially bring negative effects in patients exposed to PCSK9 inhibitors, especially concomitant with statins or with the prescription history of statins. To address this concern, apart from limiting reports submitted by health-care professionals, not drug consumers, our review further tried to address the above concern by excluding reports of PCSK9 inhibitors concomitant with statins coded as “concomitant.” This step provides additional validity and approximates more closely disproportionality. From other perspective, the number trend of the reports about PCSK9 inhibitors fluctuated year by year (311 reports in 2017, 1639 reports in 2018, and 385 reports in 2019), which may be explained, in part, by the varying population exposure to PCSK9 inhibitors.

Contrary to some common findings from RCTs and systematic reviews of RCTs [4–8] that no significant difference in serious events like myalgia has been seen between the PCSK9 inhibitors and the control group, our study has detected a risk of MAEs for PCSK9 inhibitor class monotherapy (ROR 5.92 (5.70-6.15)). Individually, alirocumab was more risky of MAEs as compared to evolocumab, and related mechanism needs in-depth exploration. The results were concordant with previous investigations [12–14], which have also detected the risk signal of muscle symptoms. In [12], ROR of muscle-related-symptoms was 1.59 (95% CI 1.44-1.75), and muscle pains was 1.51 (95% CI 1.38-1.66). It was smaller than that of our study, which may be partially attributed to the inclusion criteria. In theory, PCSK9 inhibitors are fully human monoclonal antibodies, and this characteristic can reduce the risk of immunogenicity [31]. So, the mechanism for the MAE risk for PCSK9 inhibitor needs further exploration.

### 4.3. MAEs Induced by PCSK9 Inhibitor Concomitant with Statins

More and more studies have been dedicated to clarifying the effect of concomitant statin administration on PCSK9 inhibitors' efficacy and safety [15, 16]. As we know, the target-mediated drug disposition model plays an important role in the elimination pathway for monoclonal antibodies (mAbs), where the Fab region of the antibody binds to its pharmacological target with high affinity [32, 33]. Statins can increase the target-mediated clearance of PCSK9 inhibitors and thus change the elimination rate of mAbs on the cell surface, although this change does not diminish the effect of PCSK9 inhibitors [17, 18]. A population PK (PopPK) model found that coadministration of a statin was associated with a 28~29% decrease in AUC_336_ when compared with alirocumab monotherapy [34]. According to this theory, the MAE risk for the combination of PCSK9 inhibitors and statins would be reduced because of the reduced systemic exposure of PCSK9 inhibitors.

To date, data about the MAE risk of PCSK9 inhibitors concomitant with statins is limited. Contrary to the PK/PD theory, our study found larger ROR of MAEs for the two combined drugs in clinical practice. In an open-label extension trial [19], the evolocumab plus SOC (statin or ezetimibe) group was seen with a numerically higher rate of MAEs than the group of SOC alone (10.0% vs. 4.6%). Altogether, long-term safety of concomitant use of PCSK9 inhibitors and statins deserves further careful evaluation.

### 4.4. Outcome

Five PTs were detected in our study, including myositis, myalgia, blood creatine phosphokinase increased, myopathy, and rhabdomyolysis. Population exposed to PCSK9 inhibitors would most likely tend to experience myalgia among the five MAE events. It was comparable to that of some studies [12, 35], where myalgia was the most common muscle symptoms attributed to PCSK9 inhibitors. Besides, myalgia was the common reason for drug discontinuation in clinical trials [36, 37]. For statins, rhabdomyolysis, which was more severe and even life-threatening, was the most encountered symptom. From the available data, the hospitalization rate was unsatisfactory for PCSK9 inhibitor regimens, although clinical trials have only reported discontinuation rate due to PCSK9 inhibitors [34, 35]. Thus, whenever a patient takes either PCSK9 inhibitors with or without concomitant with statin reports muscle complaints, clinicians should take PCSK9 inhibitors into account. Increased laboratory and clinical monitoring are required to timely diagnose and manage MAEs. For statin-associated MAEs, it needs more intensive care after the occurrence of muscle symptoms.

### 4.5. Limitations

This study cannot escape some limitations inherent to the study design. First, it is unable to calculate the incidence rate of AEs due to absence of denominator and the prevalence of underreporting. Second, when calculating the ROR, there exist residual confounders like basic comorbidities and comedications. Meantime, it is challenging to predict specific crucial risk factors and take prophylaxis measure to stop the occurrence of MAEs due to incomplete information [38]. Nevertheless, despite these flaws, pharmacovigilance assessments play an indispensable role in providing continued safety as drugs reach the general population.

## 5. Conclusions

Based on FAERS database between the fourth quarter of 2015 to the second quarter of 2021, this study shows that PCSK9 inhibitors are associated with MAEs, with different disproportionalities among different PCSK9 inhibitors regimens. The risk significantly increases when combined with statins. Increased laboratory and clinical monitoring are required to timely diagnose and manage MAEs. Long-term safety including MAEs about PCSK9 inhibitors should be accessed through well-designed prospective clinical trials.

## Figures and Tables

**Table 1:** Characteristics of reports of interest reported by health professionals in FAERS from 2015 quarter 4 to 2021 quarter 2 of PCSK9 inhibitors.

	PCSK9 inhibitors regimen						
	All reports	PCSK9 inhibitors monotherapy		Statin+PCSK9		Statin monotherapy	
		Cases	Noncases	Cases	Noncases	Cases	Noncases
Number	53979	3088	50502	97	292	7817	37516
*Sex*							
T\I	3 (0.01%)	—	3	—	—	1	—
Missing	2949 (5.46%)	218	2694	8	29	696	3966
Female	29386 (54.44%)	1507	27744	32	103	2956	16357
Male	21641 (40.09%)	1363	20061	57	160	4164	17193
*Age, years*							
Median	68	67	67	55	61	67	68
Interquartile range	60-75	61-74	60-74	51-65	45.25-71	58-74	59-76
0-18 years	32 (0.06%)	2	25	1	4	39	216
19-44 years	864 (1.60%)	44	781	10	29	286	1409
45-64 years	14399 (26.68%)	762	13514	44	79	2382	10041
65-74 years	14354 (26.59%)	751	13538	11	54	2130	9261
≥75 years	8871 (16.43%)	449	8375	9	38	1564	8996
Missing	15459 (28.64%)	1080	14269	22	88	1416	7593
*Reporter occupation*							
Missing	831 (1.54%)	45	781	3	2	598	2442
Pharmacist	5460 (10.12%)	304	5043	24	89	2101	10057
Physician	39214 (72.65%)	2195	36809	51	159	2996	15528
Other health professionals	8474 (15.70%)	544	7869	19	42	2122	9489
*Reporting year*							
2015	548 (1.02%)	43	505	—	—	234	1266
2016	4047 (7.50%)	359	3673	3	12	1096	5212
2017	4420 (8.19%)	311	4058	16	35	1109	4538
2018	26657 (49.38%)	1639	24929	23	66	1452	6304
2019	7684 (14.24%)	385	7239	13	47	1549	7866
2020	6915 (12.81%)	263	6546	27	79	1718	8318
2021 (Q1,Q2)	3708 (6.87%)	88	3552	15	53	659	4012
*Top 5 reporter countries*							
USA (51634, 95.65%)		USA (2896)	USA (48615)	USA (29)	USA (94)	USA (2316)	USA (10379)
Germany (394, 0.73%)		Germany (50)	Germany (289)	Germany (25)	Canada (53)	England (953)	France (6937)
Canada (390, 0.72%)		Canada (34)	Canada (284)	Canada (19)	Japan (36)	France (695)	England (4447)
Japan (281, 0.55%)		Netherlands (14)	Japan (224)	Japan (9)	Germany (30)	Germany (592)	Canada (2645)
England (204, 0.37%)		Japan (12)	England (184)	Belgium (4)	ES (15)	Italy (534)	Germany (1541)
*Outcome^$^*							
DE^#^	695 (7.36%)	5	665	—	25	366	3278
LT^#^	208 (2.20%)	6	162	1	39	582	1960
HO^#^	2977 (31.54%)	99	2807	15	56	2809	12075
DS^#^	156 (1.65%)	29	122	1	4	416	1127
CA^#^	5 (0.05%)	0	1	—	4	2	131
RI^#^	12 (0.13%)	2	10	—	—	21	16
OT^#^	5448 (57.72%)	463	4820	59	106	2341	12582
Missing^#^	44478 (82.40%)	2484	41915	21	58	1280	6347

^$^Since there are more than one outcomes of seriousness in a single report, the final level of seriousness for the single report was as the following orders: death > life threatening > hospitalization > disability > congenital anomaly > required intervention > other serious. The percentage of individual income is calculated based on the available data. ^#^DE: death; LT: life-threatening; HO: required hospitalization or prolongation of existing hospitalization; DS: disability; CA: congenital anomaly or birth defect; RI: required intervention to prevent permanent impairment/damage; OT: other serious medical events.

**Table 2:** Disproportionality analyses for the various PCSK9 inhibitor regimen and statin treatment.

DDI^∗^	Exposure	Cases	Noncases	ROR (95% CI)
PCSK9 inhibitors +statins	No PCSK9 inhibitors, no statins	39034	3777742	Reference
	PCSK9 inhibitors, no statins	3088	50502	5.92 (5.70-6.15)
	Statins, no PCSK9 inhibitors	7817	37516	20.17 (19.64-20.70)
	PCSK9 inhibitors+statins	97	292	32.15 (25.55-40.46)

Evolocumab+statins	No PCSK9 inhibitors, no statins	39034	3777742	Reference
	Evolocumab, no statins	2699	46761	5.59 (5.37-5.81)
	Statins, no evolocumab	7817	37516	20.17 (19.64-20.70)
	Evolocumab+statins	72	192	36.29 (27.68-47.59)

Alirocumab+statins	No PCSK9 inhibitors, no statins	39034	3777742	Reference
	Alirocumab, no statins	402	3976	9.79 (8.83-10.85)
	Statins, no alirocumab	7817	37516	20.17 (19.64-20.70)
	Alirocumab+statins	25	107	22.61 (14.63-34.95)

PCSK9 inhibitors +atorvastatin	No PCSK9 inhibitors, no statins	39034	3777742	Reference
	PCSK9 inhibitors, no statins	3088	50502	5.92 (5.70-6.15)
	Atorvastatin, no PCSK9 inhibitors	4217	19931	20.48 (19.78-21.20)
	PCSK9 inhibitors+atorvastatin	51	166	29.73 (21.72-40.70)

PCSK9 inhibitors +lovastatin	No PCSK9 inhibitors, no statins	39034	3777742	Reference
	PCSK9 inhibitors, no statins	3088	50502	5.92 (5.70-6.15)
	Lovastatin, no PCSK9 inhibitors	107	372	27.84 (22.45-34.52)
	PCSK9 inhibitors+lovastatin	0	29	NA^∗^

PCSK9 inhibitors +pravastatin	No PCSK9 inhibitors, no statins	39034	3777742	Reference
	PCSK9 inhibitors, no statins	3088	50502	5.92 (5.70-6.15)
	Pravastatin, no PCSK9 inhibitors	606	2801	20.93 (19.17-22.87)
	PCSK9 inhibitors+pravastatin	22	29	73.42 (42.18-127.80)

PCSK9 inhibitors +rosuvastatin	No PCSK9 inhibitors, no statins	39034	3777742	Reference
	PCSK9 inhibitors, no statins	3088	50502	5.92 (5.70-6.15)
	Rosuvastatin, no PCSK9 inhibitors	2079	8090	24.87 (23.68-26.13)
	PCSK9 inhibitors+rosuvastatin	48	136	34.16 (24.58-47.48)

PCSK9 inhibitors +simvastatin	No PCSK9 inhibitors, no statins	39034	3777742	Reference
	PCSK9 inhibitors, no statins	3088	50502	5.92 (5.70-6.15)
	Simvastatin, no PCSK9 inhibitors	2022	7485	26.14 (24.87-27.49)
	PCSK9 inhibitors+simvastatin	24	66	35.19 (22.06-56.16)

PCSK9 inhibitors +pitavastatin	No PCSK9 inhibitors, no statins	39034	3777742	Reference
	PCSK9 inhibitors, no statins	3088	50502	5.92 (5.70-6.15)
	Pitavastatin, no PCSK9 inhibitors	122	422	26.14 (24.87-27.49)
	PCSK9 inhibitors+pitavastatin	7	9	75.27 (28.03-202.13)

PCSK9 inhibitors +fluvastatin	No PCSK9 inhibitors, no statins	39034	3777742	Reference
	PCSK9 inhibitors, no statins	3088	50502	5.92 (5.70-6.15)
	Fluvastatin, no PCSK9 inhibitors	59	346	16.50 (12.52-21.75)
	PCSK9 inhibitors+fluvastatin	0	1	NA^∗^

^∗^Drug-drug interaction: DDI. NA: not applicable because of incapability to calculate the ROR (the absence of cases).

**Table 3:** Outcome distribution in terms of each PT^$^ for cases with MAEs^$^ because of PCSK9 inhibitors and statins.

	DE^#^	LT^#^	HO^#^	DS^#^	CA^#^	RI^#^	OT^#^	*N*%
PCSK9 inhibitors +statins								
Rhabdomyolysis		1	4				6	12.09%
Blood CPK^∗^ increased			1				15	17.58%
Myalgia			11	1			51	69.23%
Myositis							1	1.10%
*N*%	0.00%	1.10%	17.58%	1.10%	0.00%	0.00%	80.22%	
PCSK9 inhibitor monotherapy								
Myalgia	4	5	83	31		3	438	80.92%
Rhabdomyolysis	1	1	10				30	6.03%
Myopathy			2	2			9	1.87%
Blood CPK^∗^ increased			12				58	10.04%
Myositis			2				6	1.15%
*N*%	0.72%	0.86%	15.64%	4.73%	0.00%	0.43%	77.62%	
Statins monotherapy								
Blood CPK^∗^ increased	24	104	620	74	1	11	759	14.07%
Rhabdomyolysis	285	490	2298	138	3	45	1789	44.59%
Myopathy	20	70	376	95	1	8	540	9.81%
Myositis	12	25	140	25		4	182	3.43%
Myalgia	59	137	735	406		33	1811	28.10%
*N*%	3.53%	7.30%	36.83%	6.52%	0.04%	0.89%	44.89%	

^$^PT: preferred term; MAEs: musculoskeletal adverse events; ^∗^CPK: creatine phosphokinase; ^#^DE: death; LT: life-threatening; HO: required hospitalization or prolongation of existing hospitalization; DS: disability; CA: congenital anomaly or birth defect; RI: required intervention to prevent permanent impairment/damage; OT: other serious medical events.

## Data Availability

Data are available in https://www.fda.gov/drugs/questions-and-answers-fdas-adverse-event-reporting-system-faers/fda-adverse-event-reporting-system-faers-public-dashboard.

## Supplementary Materials

Supplementary Materials Table 1: search terms for drug names of statins and PCSK9 inhibitors used in this study. Table 2: preferred terms for identifying of musculoskeletal adverse events by SMQ.
