# Supplementary material for: Musculoskeletal Adverse Events Associated with PCSK9 Inhibitors: Disproportionality Analysis of the FDA Adverse Event Reporting System
Source: Cardiovasc Ther. 2022 Jan 25;2022:9866486. doi: 10.1155/2022/9866486 (PMC8808238; doi:10.1155/2022/9866486)
Supplement: Supplementary Materials — Table 1: search terms for drug names of statins and PCSK9 inhibitors used in this study. Table 2: preferred terms for identifying of musculoskeletal adverse events by SMQ. [file 9866486.f1.docx]

**Supplementary Table 1 Search terms for drug names of statins and PCSK9 inhibitors used in this study**

| Statins | Atorvastatin | Atorvastatin  LIPITOR  TAHOR  TORVAST  SORTIS  TOTALIP  ATORVASTIN  ATORIS  Caduet |
| --- | --- | --- |
|  | Lovastatin | Lovastatin  MEVACOR  LOVASTIN  ALTOPREV  LOVASTATINE |
|  | Pravastatin | Pravastatin  PRAVACHOL  MEVALOTIN  ELISOR  PRAVACOL  VASTEN  PRAVASIN  LIPOVAS |
|  | Rosuvastatin | Rosuvastatin  CRESTOR  TREZOR  PROVISACOR  ROSUCOR  ZUVAMOR  TREZOR  RUSOVAS |
|  | Simvastatin | Simvastatin  ZOCOR  SIMVASTIN  SIMVAHEXAL  SINVASTATINA  SINVASCOR |
|  | Fluvastatin | Fluvastatin  Lescol  Lescol XL  [Canef](https://www-micromedexsolutions-com.autorpa.cych.org.tw/micromedex2/librarian/CS/8122E7/ND_PR/evidencexpert/ND_P/evidencexpert/DUPLICATIONSHIELDSYNC/E2CACB/ND_PG/evidencexpert/ND_B/evidencexpert/ND_AppProduct/evidencexpert/ND_T/evidencexpert/PFActionId/evidencexpert.IntermediateToDocumentLink?docId=8546048&contentSetId=62&currentItemOnPage=5)  Flustatin  Flutisan  Lochol |
|  | Pitavastatin | Pitavastatin  Zypitamag  Livalo  Nikita  Lester  Pitalip  Pitator  Pitava  Pivast  Pratin |
| PCSK9 inhibitors | Evolocumab | Evolocumab  Repatha |
|  | Alirocumab | Alirocumab  Praluent |

**Supplementary Table 2 Preferred terms for identifying of musculoskeletal adverse events by SMQ**

| Preferred terms |  | Preferred terms |
| --- | --- | --- |
| **blood creatine phosphokinase increased** |  | **myalgia** |
| CK increased |  | generalised muscle aches |
| CPK increase |  | generalized muscle aches |
| CPK increased |  | localised muscle aches |
| creatine kinase high |  | localized muscle aches |
| creatine kinase increased |  | muscle ache |
| creatine phosphokinase increased |  | muscle burning sensation |
| creatine phosphokinase serum increased |  | muscle pain |
| phosphokinase creatine serum increased |  | muscle soreness |
| plasma creatine phosphokinase increased |  | muscle tenderness any site |
| serum creatine phosphokinase increased |  | muscular pain |
|  |  | muscular pains |
| **myopathy** |  | myalgia |
| acute myopathy |  | myalgia aggravated |
| axial myopathy |  | myalgia of lower extremities |
| myopathy |  | myalgia of upper extremities |
| myopathy aggravated |  | pain muscle |
| myopathy steroid |  | polymyalgia |
| myopathy steroid-induced |  | polymyalgia aggravated |
| myopathy unspecified |  | polymyalgia worsened |
| proximal myopathy |  | tenderness muscle |
| proximal myopathy aggravated |  |  |
| steroid myopathy |  | **rhabdomyolysis** |
| symptomaticc inflammatory myopathy |  | muscle dissolution |
|  |  | rhabdomyolysis |
| **muscle spasm** |  |  |
| back muslce spasm |  | **myositis** |
| cervical spasm |  | granulomatous myositis |
| charley horse |  | interstitial myositis |
| cramp |  | muscle inflammation |
| cramp in hand |  | myositis |
| cramp legs |  | myotenositis |
| cramp muscle |  | traumatic myositis ossificans |
| cramp of limb |  |  |
| cramps |  |  |
| cramps calf |  |  |
| cramps in legs |  |  |
| cramps in the calves |  |  |
| cramps legs |  |  |
| cramps leg |  |  |
| cramps of extremities |  |  |
| crampsof lower body |  |  |
